# Separation and Determination of Paraquat and Diquat in Human Plasma and Urine by Magnetic Dispersive Solid Phase Extraction Coupled with High-Performance Liquid Chromatography

**DOI:** 10.1155/2020/7359582

**Published:** 2020-07-16

**Authors:** Ou Sha, Bowen Cui, Xiaobing Chen, Hua Liu, Jiawei Yao, Yuqing Zhu

**Affiliations:** ^1^Department of Chemical Engineering, Jiangsu Ocean University, Lianyungang 222005, China; ^2^Analysis and Test Center of Jiangsu Marine Resources Development Research Institute, Jiangsu, Lianyungang 222005, China; ^3^The First People's Hospital of Lianyungang, Lianyungang 222005, China

## Abstract

A magnetic dispersive solid phase extraction method coupled with high-performance liquid chromatography was proposed for the simultaneous separation and determination of paraquat (PQ) and diquat (DQ) in human plasma and urine samples. Based on the reduction of PQ and DQ to a blue radical and yellow-green radical by sodium dithionite in an alkaline medium, a fast colorimetric method was also developed for the fast detection of PQ or DQ. In this paper, CoFe_2_O_4_@SiO_2_ magnetic nanoparticles were used as the adsorbent for the magnetic dispersive solid phase extraction of paraquat and diquat, and these two analytes were found to be eluted directly from the adsorbent by NaOH solution. The main factors affecting the extraction efficiency including amount of extractant, extraction time, sample volume, sample solution pH, and elution volume were optimized. Under the optimized experimental conditions, the calibration curve was linear at a concentration range of 28.5–570.2 *μ*g/L, and the correlation coefficient of paraquat and diquat was 0.9986 and 0.9980, respectively. The limits of detection of paraquat and diquat were 4.5 *μ*g/L and 4.3 *μ*g/L. The proposed MSPE-HPLC method was successfully applied to the detection of the paraquat and diquat in human plasma and urine with satisfied recoveries of PQ and DQ in the range of 87.5%–98.7%.

## 1. Introduction

**Figure 1:**
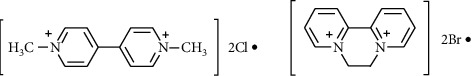
The structure of PQ and DQ.

Paraquat (PQ) and diquat (DQ) are nonselective contact herbicides, which have been widely used in the world for controlling the growth of weeds and grass in order to achieve high agricultural productivity (Figure 1) [1, 2]. However, PQ is highly toxic to human, and an exposure to paraquat via accidental or suicidal ingestion can cause acute renal toxicity and be frequently fatal in small doses. Compared with PQ, DQ causes severe toxic effect on the central nervous system [3]. Although PQ has been banned by the European Union and China and will be banned in Malaysia and other developing countries in the future, it is ubiquitous and can still be obtained in the developing countries for the characteristics of lower price and the most cost-effective control of weed [4, 5]. Findings are reported by Mai that more than 300 PQ-poisoned cases reported per year in Vietnam [6].

Usually the concentrations of PQ or DQ in plasma and urine are applied to diagnose the severity of poisoning and help doctors to rescue patients [7]. Different approaches to measuring PQ or DQ concentration are available including high-performance liquid chromatography (HPLC) [8], high-performance liquid chromatography-mass spectrometry (HPLC–MS) [9], gas chromatography-mass spectrometry (GC–MS) [3], square wave voltammetry [10], and the spectrophotometric method [11]. Another option reported in the literature is the application of surface-enhanced Raman spectroscopy (SERS) [12]. Compared with other methods, HPLC is most commonly used for determination of PQ and DQ because the instrument is commonly available in laboratories. And HPLC has high sensitivity and separation efficiency.

Since PQ and DQ are both highly soluble in water and have extremely poor affinity for nonpolar solvents, making the separation and preconcentration before the determination of PQ or DQ is often problematic. The complexity, time consumption, and equipment demand of most approaches makes them impractical, especially in resource-poor environments. Improved sample preparation approaches for the extraction of PQ or DQ from plasma and urine samples, such as solid phase extraction [13], liquid phase extraction [14], and so on, have been developed [15]. However, the complex procedure including protein precipitation [16] by acetonitrile or protein removal by ultrafiltration membrane could reduce the sensitivity of the detection method and increase the analysis time.

Magnetic dispersive solid phase extraction (MDSPE) is a promising sample separation and pretreatment technique. In MDSPE, the magnetic adsorbent is dispersed in sample solution containing the target analytes. The higher the interfacial area between the extractant and sample, the faster the mass transfer, thus equilibrium is reached sooner. After extraction, an external magnetic field was applied to separate the enriched magnetic sorbent, and the sorbent phases with magnetic properties enable assisted magnetic separation of the aqueous sample. This approach enables the sorbent to interact equally with all the sorbent particles, achieving greater capacity per amount of sorbent and avoiding channeling or blocking of cartridges or disks, as it occurs in traditional SPE [17, 18].

CoFe_2_O_4_, as an excellent soft magnetic material, has good superparamagnetism with high saturation magnetization and anisotropy constant, and it can be assembled into tiny and multifunctional biocompatible devices [19]. Aygar et al. synthesized CoFe_2_O_4_@SiO_2_ nanoparticles for the purification of histidine-tagged proteins [20]. Abdolmohammad-Zadeh et al. prepared CoFe_2_O_4_ nanoparticles functionalized with 8-hydroxyquinoline for dispersive solid-phase microextraction and direct fluorometric monitoring of aluminum in human serum [21].

Depending on the urgent needs for developing rapid and high selective methods to determine PQ and DQ with lower detection limits in plasma and urine samples, magnetic dispersive solid phase extraction coupled with HPLC-UV, CoFe_2_O_4_@SiO_2_ nanoparticles selected as the adsorbent, was developed for the separation and determination of PQ and DQ for the first time. After a series of optimization of extraction conditions, the proposed analytical method with notably reduced matrix effects was successfully applied for determination of PQ and DQ. It was contributed to reduce the matrix effects with the procedure of no need of protein precipitation and protein removal. Meanwhile, based on the reduction of PQ and DQ to form a blue radical and yellow-green radical by sodium dithionite in alkaline medium, respectively, a rapid and simple method was established to colorimetrically detect PQ or DQ in human plasma and urine samples with naked eyes. This method proposed in this paper could offer a fast assessment of patient outcome based on the experimental data and helped the doctor to decide treatment strategies.

## 2. Experimental

### 2.1. Materials and Apparatus

Paraquat and diquat were purchased from the Chinese National Institute for the Control of Pharmaceutical and Biological Products (Beijing, China). Iron nitrate nonahydrate (Fe(NO_3_)_3_·9H_2_O), cobaltous nitrate hexahydrate (Co(NO_3_)_2_·6H_2_O), sodium citrate (Na_3_C_6_H_5_O_7_), sodium dithionite (Na_2_S_2_O_4_), tetramethyl orthosilicate (TEOS), polyethylene glycol 6000 (PEG 6000), phosphoric acid (H_3_PO_4_), ethanol, and triethylamine were purchased from Sinopharm Chemical Reagent Co., Ltd (Shanghai, China). The stock solution of PQ or DQ (100.0 mg/L) was prepared by dissolving the appropriate amount of reagent in deionized water and storing at 4°C in the refrigerator until used. Solutions with lower paraquat or diquat concentrations were made by appropriate dilution of the standard solution. Britton–Robinson (B–R buffer) solution is configured with 0.04 mol/L mixed acid (H_3_PO_4_, HAc, and H_3_BO_3_) and 0.2 mol/L NaOH solution. All reagents were of analytical grade.

### 2.2. Instrument

The HPLC analysis was performed by LC-20AD chromatograph (Shimadzu Corporation, Japan). The pH value was adjusted by a PHS-25B pH meter (Shanghai Precision & Scientific Instrument Co., Ltd., Shanghai, China). X-ray diffraction (XRD) pattern was collected by a X'Pert Pro diffractometer (PANalytical, Netherlands). The Fourier transform infrared spectra (FT-IR) were recorded on a Bruker Vector 22 spectrometer (Brook company, Germany). Magnetization measurement was studied by an MPMS-7 vibrating sample magnetometer (VSM) (Quantum Design, USA).

### 2.3. Synthesis of CoFe_2_O_4_@SiO_2_ MNPs

Synthesis of CoFe_2_O_4_@SiO_2_ was carried out using the Stӧber method [22]. 5.82 g Co(NO_3_)_2_·6H_2_O, 16.16 g Fe(NO_3_)_3_·9H_2_O, and 5.88 g Na_3_C_6_H_5_O_7_ were dispersed in 100 mL of deionized water. Then, 30 mL of NH_3_·H_2_O was quickly added into the solution to obtained the gels under stirring at 80°C for 2 h. The gels were thermally treated at 500°C for 3 h to obtain CoFe_2_O_4_.

Then, 2.0 g of CoFe_2_O_4_ was dissolved in distilled water and placed in a three-neck flask in an ultrasonic bath for 10 min and dispersed in 40.0 mL of distilled water, 80 mL of ethanol, and 5.0 mL of ammonia. After that, 2.0 mL of TEOS was added slowly to suspension under mechanical stirring. The reaction was allowed to proceed at 60°C for 12 h. The obtained *c* was washed three times with deionized water and ethanol, respectively, and dried at 60°C for 6 h.

### 2.4. Sample Collection

This study was approved by the Ethics Committee of the Jiangsu Ocean University (Lianyungang, China). Human plasma samples were obtained from patients of PQ intoxication and healthy human with no known record of occupational exposure to PQ (as blank) from the First People's Hospital of Lianyungang. Written consent was signed by all participants. Plasma samples spiked with standard PQ stock solutions to indicate concentrations were used as positive controls. The venous blood was drawn from the forearm of subjects and rapidly transferred to vessels containing heparin as an anticoagulant. Plasma was separated by centrifugation of the venous blood at 14000 rpm for 10 min and stored at 4°C. Human urine samples were obtained from the First People's Hospital of Lianyungang and stored at 4°C. The urine samples were centrifuged for 30 min at 3000 rpm and filtered through 0.45 *μ*m membrane filters, and no further treatment was needed.

### 2.5. Quantitative Process

**Figure 2:**
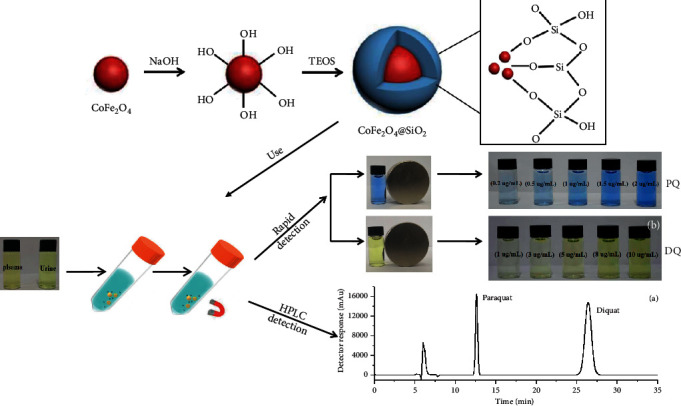
Preparation of CoFe_2_O_4_@SiO_2_ and the MSPE procedure for paraquat/diquat in plasma and urine.

Human plasma or urine samples were added into a 40 mL polyethylene tube and diluted to the scale line with deionized water. Then, 30.0 mg CoFe_2_O_4_@SiO_2_ MNPs were put into this solution and dispersed by vortex for 10 min. The MNPs were collected by a Nd-Fe-B magnet, whereas the supernatant solution was discarded. The collected MNPs were washed three times by deionized water, then immersed into 1.0 mL of 2 mol/L NaOH, and vortexed for 1 min. Magnet was used to separate the MNPs again. Then, the eluent was adjusted to pH 7.0 with phosphoric acid and filtered through a 0.22 *μ*m nylon syringe filter. A total 20 *μ*L of elution was injected into the HPLC system for DQ or PQ determination. The complete process of MDSPE system combined with HPLC–UV for determination of PQ and DQ was shown in Figure 2. The extraction efficiency (1) and the amount of analyte adsorbed per unit mass of the adsorbent (adsorption capacity) (2) were calculated by the following equation:

(1) $$\begin{array}{c} \text{Extraction efficiency }\left(\text{\%}\right)=\frac{C_{0}-C_{t}}{C_{0}}\times 100\%, \end{array}$$

(2) $$\begin{array}{c} \text{Adsorption capacity}=\frac{C_{0}-C_{t}}{m}\times V. \end{array}$$

where *C*_*t*_ (*μ*g/mL) was the concentration of PQ or DQ in the supernatant after adsorption; *C*_0_ (*μ*g/mL) was the initial concentration of PQ or DQ; *V* (L) was the volume of sample solution; and *m* (g) was the mass of adsorbents.

### 2.6. Colorimetric Detection Process

The colorimetric detection of paraquat or diquat concentration was performed by comparing the color against the standard colorimetric systems, which were made with known concentrations of PQ and DQ. After the MDSPE extraction procedure, as described in subsection 2.4, 25.0 mg/L of Na_2_S_2_O_4_ solution (0.5 mL) was added to the eluent of PQ or DQ. Then, observation was done after 2 minutes shaking time and 1 minute stand-by time. The colored solutions corresponding to different target concentrations were finally measured by naked eyes for the colorimetric detection.

### 2.7. HPLC Detection Conditions

Welch AQ-C18 column (4.6 mm × 250 mm, 5 *μ*m, USA), an analytical column, was used for analyte. The mobile phase was a mixture of methanol and 0.2 mol/L sodium dihydrogen phosphate buffer (10 : 90, v/v) containing both 0.1 mol/L triethylamine and 12 mmol/L sodium 1-heptane sulfonate. The mobile phase was adjusted to pH 2.8 with phosphate acid at a flow rate of 0.5 mL·min^−1^. The injection volume was 20 *μ*L, and the detection wavelengths of PQ and DQ were set at 258 nm and 310 nm, respectively.

## 3. Results and Discussion

### 3.1. Characterization of CoFe_2_O_4_@SiO_2_

**Figure 3:**
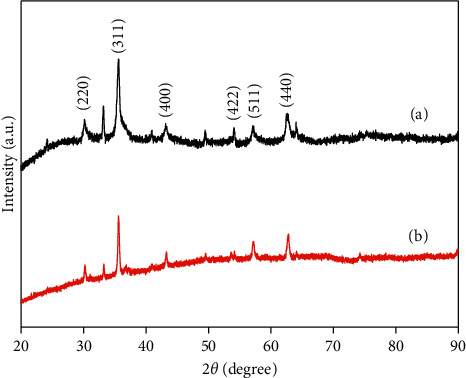
XRD patterns of CoFe_2_O_4_@SiO_2._ (a) and CoFe_2_O_4_ (b).

The XRD patterns of CoFe_2_O_4_@SiO_2_ (a) and CoFe_2_O_4_ (b) are shown in Figure 3. It can be observed that the XRD pattern of CoFe_2_O_4_ MNPs (curve (b)) is consistent with the ICPDS file of CoFe_2_O_4_ (No.22–1086) [23]. And the diffraction peaks of CoFe_2_O_4_@SiO_2_ (curve (a)) are similar to those of CoFe_2_O_4_. Compared with these two curves, the broad peak at 20–28° found in CoFe_2_O_4_@SiO_2_ correspond to the silica coated on the surface of CoFe_2_O_4_, which corresponds to the standard diffraction pattern of SiO_2_ [24]. The result illustrated that the SiO_2_ was successfully coated on the surface of CoFe_2_O_4_ MNPs.

**Figure 4:**
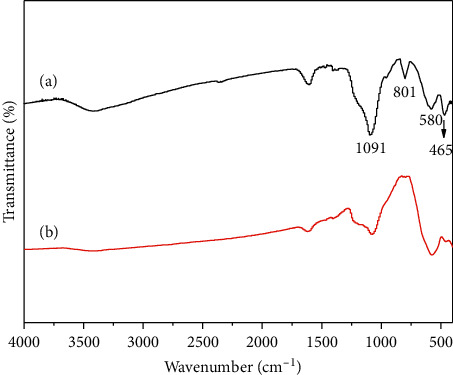
FT-IR spectra of CoFe_2_O_4_@SiO_2_ (a) and CoFe_2_O_4_ (b).

The functional groups and chemical bonds in CoFe_2_O_4_@SiO_2_ (curve (a)) and CoFe_2_O_4_ (curve (b)) were analyzed by means of FT-IR. The diffraction peaks of 465 cm^−1^, 778 cm^−1^, and 1130 cm^−1^ attributed to the bending vibrations of the Si-O-Si are shown in Figure 4. The presence of Fe-O bonds in the magnetic particles was confirmed by the characteristic peaks appeared at 580 cm^−1^ [25]. These results revealed the successful preparation of CoFe_2_O_4_@SiO_2_ MNPs.

**Figure 5:**
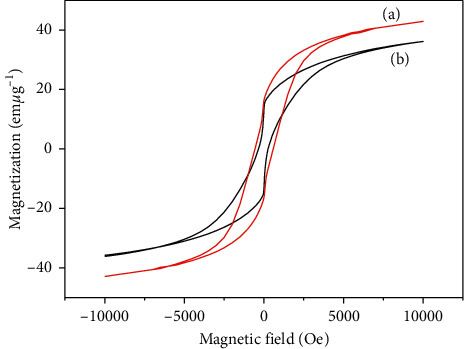
Magnetic hysteresis curves of CoFe_2_O_4_ (a) and CoFe_2_O_4_@SiO_2_ (b).

The magnetic properties of CoFe_2_O_4_@SiO_2_ and CoFe_2_O_4_ were elucidated by the magnetization hysteresis curve. As shown in Figure 5, the saturation magnetization values of the CoFe_2_O_4_@SiO_2_ and CoFe_2_O_4_ were 36 emu g^−1^ and 42 emu g^−1^, respectively. The saturation magnetization of CoFe_2_O_4_@SiO_2_ was lower than CoFe_2_O_4_, which was attributed to the effect of the amorphous silica layer coated [26]. Although the saturation magnetization of CoFe_2_O_4_ decreased after coating SiO_2_, the CoFe_2_O_4_@SiO_2_ and CoFe_2_O_4_ could be rapidly separated from the solution by Nd-Fe-B magnet.

### 3.2. Optimization of PQ and DQ Extraction Conditions

To investigate the optimum conditions for the MDSPE process of PQ and DQ, 30.0 mg of CoFe_2_O_4_@SiO_2_ was spiked with the mixture of PQ or DQ at a concentration of 500 *µ*g/L. The relevant experimental conditions including pH value, amount of adsorbent, extraction time, sample volume, and elution volume were optimized. All the experiments were performed in triplicates.

#### 3.2.1. Effect of pH

**Figure 6:**
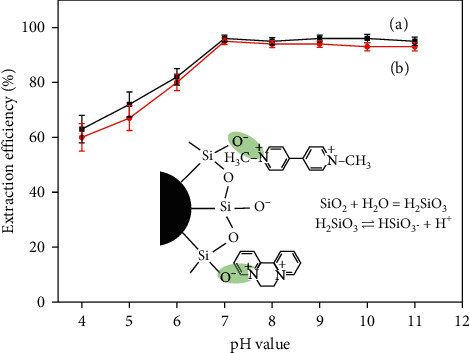
Effect of pH for the extraction efficiency of PQ (a) and DQ (b) and the adsorption mechanism.

The pH of the sample solution plays an important role in the extraction of PQ and DQ by affecting both the existing forms of the target compounds and the adsorbent. In this study, the effect of sample pH ranging between 4.0 and 11.0 was investigated by adding appropriate volumes of B–R buffer solution. As shown in Figure 6, the extraction efficiencies of PQ and DQ were increased by the pH and kept stable in the pH range of 7.0–11.0. Generally, paraquat and diquat are used as the forms of dichlorides and dibromides. In aqueous solution, they are both positively charged form and are retained stable when the solution is under weak acid or neutral conditions, whereas they are unstable in alkaline solution with a high pH value [27]. When CoFe_2_O_4_@SiO_2_ nanoparticles were selected as the sorbent, it has been reported that the surface negative charge of CoFe_2_O_4_@SiO_2_ magnetic nanoparticles could be changed with the pH of the solution. As the pH of the solution increases, the negative charge on the surface of the nanoparticles increases gradually. Therefore, the cationic herbicide could be adsorbed to the adsorbent surface by an electrostatic interaction with the CoFe_2_O_4_@SiO_2_ magnetic nanoparticles [28].

**Figure 7:**
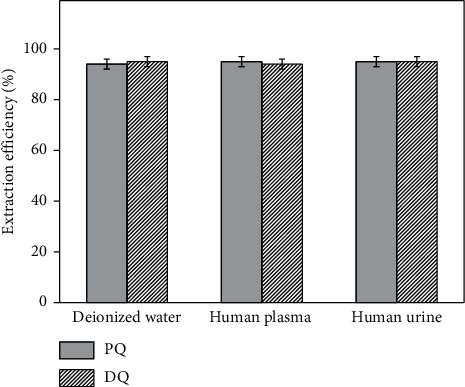
The extraction efficiencies of PQ and DQ in different samples.

Since the pH value of body fluids generally fluctuates between 6.8 and 7.5, pH 7.0 was selected for further experiments. The extraction efficiencies of PQ and DQ in different samples are shown in Figure 7. It was found that the matrix effects on the extraction efficiency of PQ and DQ could be neglected. Therefore, a standard solution was chosen for the optimization process.

#### 3.2.2. Effect of the Amount of Extractant

**Figure 8:**
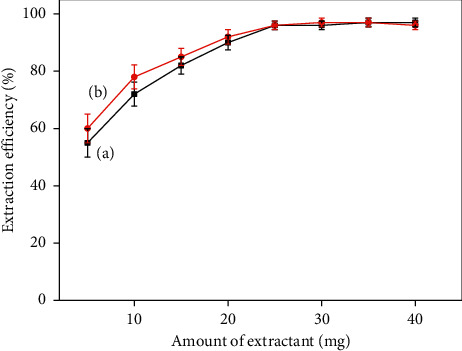
Effect of the amount of adsorbent for the extraction efficiency of PQ (a) and DQ (b).

As the amount of adsorbent increases, it can provide a larger adsorption specific surface area, thus increasing the extraction rate. To select the optimal amount of the magnetic extractant for extraction of the PQ/DQ, the different amount of CoFe_2_O_4_@SiO_2_ MNPs was evaluated in the range of 1.0 mg–40.0 mg. As shown in Figure 8, the extraction efficiency of PQ/DQ increased significantly with the amount of CoFe_2_O_4_@SiO_2_ MNPs in the range from 1.0 mg to 25.0 mg. Subsequently, the maximum extraction efficiency was achieved and kept stable while the amount of adsorbent was above 25.0 mg. Therefore, 30.0 mg of extractant was selected for further experiments.

#### 3.2.3. Effect of Extraction Time

**Figure 9:**
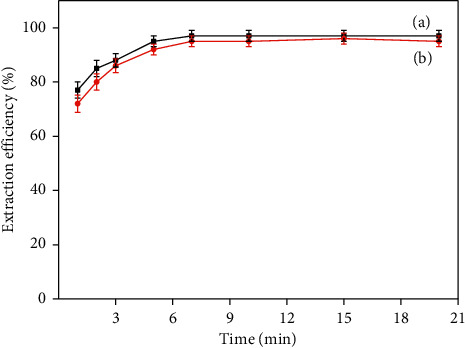
Effect of time for the extraction efficiency of PQ (a) and DQ (b).

In the MDSPE process, the extraction time is one of the critical factors that influence the extraction efficiency of PQ and DQ. The effect of extraction time on the extraction efficiency was investigated in the range of 0–20 min. With the increased extraction time, the contact between the extractant and the analyte was more effective, and accordingly, the adsorption equilibrium was promoted. As shown in Figure 9, the maximum extraction efficiency was obtained and remained constant while extraction time exceeded 10 min. Therefore, the extraction time of 10 min was selected for further experiment.

#### 3.2.4. Effect of Sample Volume

**Figure 10:**
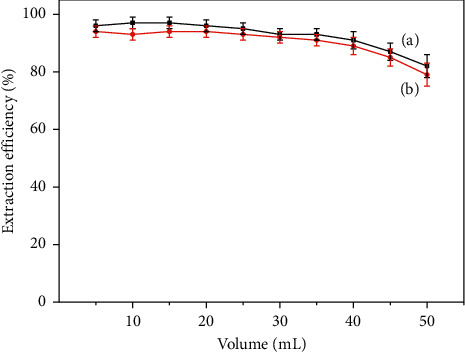
Effect of volume for the extraction efficiency of PQ (a) and DQ (b).

Preconcentration factor is a significant factor for the analysis method. The effect of sample volume on extraction efficiency was investigated by changing the sample volumes. As shown in Figure 10, the extraction efficiency remained stable (*E* ≥ 90%) when the sample volume was less than 40.0 mL. Then, the extraction efficiency was reduced obviously. Since the extraction time and the amount of adsorbents were set at a certain value, the dispersion degree of adsorbents in sample solution decreased with the increase of sample volume, resulting in the decrease of effective contact probability between the adsorbent and the analytes. Therefore, the extraction efficiency reduced, whereas the preconcentration factor of analytes increased by increasing the sample volume. Therefore, 40.0 mL was selected as the maximum sample volume to obtain a higher preconcentration factor and the better extraction efficiency.

#### 3.2.5. Adsorption Capacity

**Figure 11:**
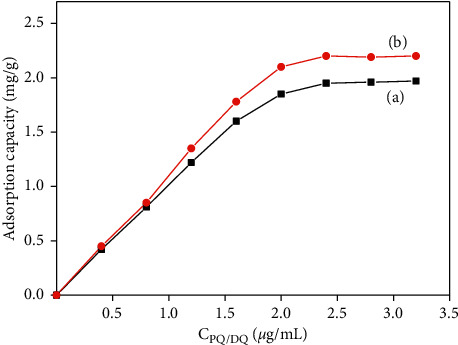
Adsorption capacity of CoFe_2_O_4_@SiO_2_ to PQ (a) and DQ (b).

Adsorption capacity is a significant parameter for adsorption properties. In this work, the adsorption capacity of CoFe_3_O_4_@SiO_2_ MNPs for PQ and DQ was calculated by varying the concentration of PQ or DQ, while the amount of extractant was selected as 30.0 mg, and the sample volume was selected as 20.0 mL. As shown in Figure 11, the adsorption capacity for PQ and DQ reached 1.8 mg/g or 2.2 mg/g, while the concentration of PQ or DQ was 2.5 mg/L.

#### 3.2.6. Effect of Elution Volume

Although PQ and DQ could be absorbed by CoFe_2_O_4_@SiO_2_ MNPs under the pH of 7.0–12.0, the reduced product of PQ and DQ reacted with the strong alkaline (NaOH solution) could not be absorbed by CoFe_2_O_4_@SiO_2_ MNPs [29]. Therefore, 2.0 mol/L of NaOH solution was chosen as the eluent. The effect of the elution solvent volume was investigated in the range of 0.2–2.0 mL. When the volume of eluent increased to 1.0 mL, PQ and DQ could be desorbed completely. To obtain a higher preconcentration factor, 1.0 mL of desorption solvent volume was chosen.

#### 3.2.7. Reusability Test

**Figure 12:**
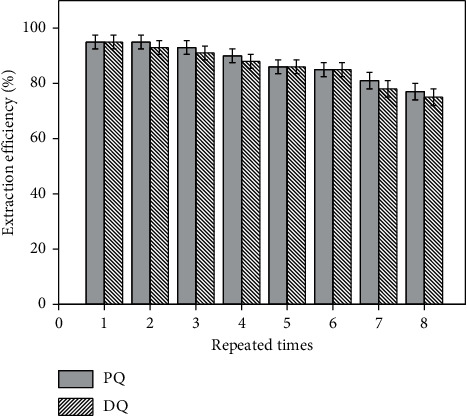
Reusability tests of CoFe_2_O_4_@SiO_2_ MNPs for the extraction efficiency toward PQ/DQ.

The adsorbed CoFe_2_O_4_@SiO_2_ MNPs were washed with 2 mol/L NaOH solution and distilled water, respectively, and then, the regenerated MNPs were used for the adsorption of PQ or DQ again. As shown in Figure 12, the adsorption efficiencies of PQ and DQ are still maintaining 85% after six times adsorption/desorption procedures, which illustrated that CoFe_2_O_4_@SiO_2_ MNPs have a better recycling performance for extraction of PQ and DQ.

#### 3.2.8. Adsorption and Desorption Mechanisms

**Figure 13:**
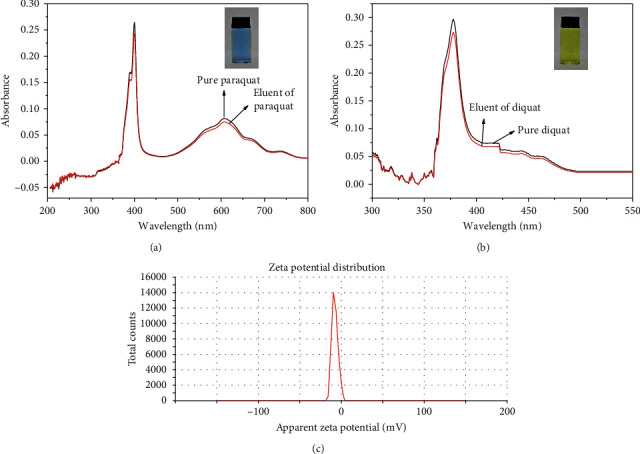
(a) The UV spectrogram of PQ-NaOH-Na_2_S_2_O_4_ and eluent-Na_2_S_2_O_4_; the insert image is blue free radicals of paraquat. (b) The UV spectrogram of DQ-NaOH-Na_2_S_2_O_4_ and eluent-Na_2_S_2_O_4_; the insert image is green free radicals of diquat. (c) The zeta potential of CoFe_2_O_4_@SiO_2_ NPs.

In this study, the adsorption and desorption mechanisms of CoFe_2_O_4_@SiO_2_ toward PQ/DQ were investigated. As the result of zeta potential, the surface of CoFe_2_O_4_@SiO_2_ NPs had negative charge with a zeta potential of −8.1 mV, which generated the electrostatic attraction with cationic pesticides (PQ or DQ). Therefore, the adsorption of CoFe_2_O_4_@SiO_2_ to PQ/DQ could be ascribed to electrostatic attraction. Depending on previous reports, paraquat and diquat can be reduced to blue free radicals and green free radicals by Na_2_S_2_O_4_ in an alkaline solution [30]. In order to prove that PQ and DQ can be desorbed by NaOH solution, Na_2_S_2_O_4_ was added to the eluents of PQ and DQ, respectively. The UV spectrogram of PQ/DQ-NaOH-Na_2_S_2_O_4_ and eluent of PQ/DQ-Na_2_S_2_O_4_ are shown in Figure 13. No noticeable differences were found between these two spectral curves. The results revealed that PQ and DQ could be desorbed completely from the surface of CoFe_2_O_4_@SiO_2_ by NaOH solution.

### 3.3. Colorimetric Detection Method

**Figure 14:**
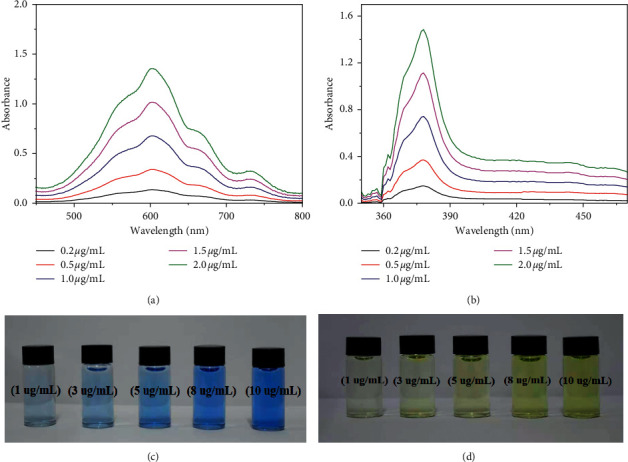
The UV spectra of PQ (a) and DQ (b) at different concentrations from 0.2 to 2.0 *μ*g/mL. The colorimetric responses to different concentrations of PQ (c) and DQ (d) from 0.2–2.0 *μ*g/mL.

In aqueous systems, paraquat and diquat could be reduced to form blue radicals and yellow radicals by sodium dithionite in an alkaline medium, respectively. Under the optimum conditions, the working range concentration was studied by using different concentrations of PQ and DQ from 0.2 to 2.0 *μ*g/mL. The developed color of these two radical was observed by the naked eye. The color change depended on the amount of PQ and DQ as shown in Figure 14. The gradual change to the blue and the yellow color was observed by the naked eye with the increased concentration. At the concentration of 0.2 *μ*g/mL of PQ or DQ, the obtained color can be distinguished from blank by the naked eye. So the detection limits of this colorimetric detection method were both 0.2 *μ*g/mL for PQ and DQ in human plasma (5 mL) and both 0.02 *μ*g/mL in human urine sample (50 mL).

### 3.4. Analytical Properties

Under the optimal conditions, a series of quantitative parameters with regard to the linearity, limits of detection (LODs), limit of quantitation (LOQ), and the preconcentration factor were used to validate the proposed MDSPE–HPLC method. Linear regression analysis was performed using peak areas against the concentrations of PQ and DQ. The good linearity was obtained in the range of 28.5–570.2 *μ*g/L for PQ and DQ with good correlation coefficients (>0.9950). The LOD and LOQ defined as three times and ten times ratio of signal to noise for chromatograms were obtained from the blank, respectively [31]. The LOD of PQ and DQ was 4.5 *μ*g/L and 4.3 *μ*g/L, respectively. The LOQ of PQ and DQ was 15.2 *μ*g/L and 14.3 *μ*g/L, respectively. The preconcentration factor, defined as the slope of the regression equations before the extraction and after the elution, was 30.8 fold.  Table 1 presents a comparison of the performance of the proposed MDSPE method with other methods for determination of PQ in plasma and urine. The proposed method described herein provided a lower range of LOD and satisfactory recoveries compared to other reported methods.

### 3.5. Precision and Accuracy

Quantification of paraquat and diquat in human plasma and urine was evaluated for both intraday and interday precision and accuracy at three different concentration levels with relative standard deviation (RSD). Intraday precision and accuracy were evaluated with 6 replicate bench preparations at each concentration level analyzed on the same day. Interday precision and accuracy were evaluated with a total of 18 replicate bench preparations in three consecutive days.  Table 2 indicated the recoveries of the spiked concentrations (10.0, 20.0, and 50.0 ng/mL) of PQ or DQ in plasma and urine samples. The intraday recovery ranged from 88.6% to 96.7% with the RSD varied from 2.7% to 5.8%. The interday recovery ranged from 84.9% to 92.7% with the RSD varied from 2.6% to 6.7%.

### 3.6. Application of Real Sample

**Figure 15:**
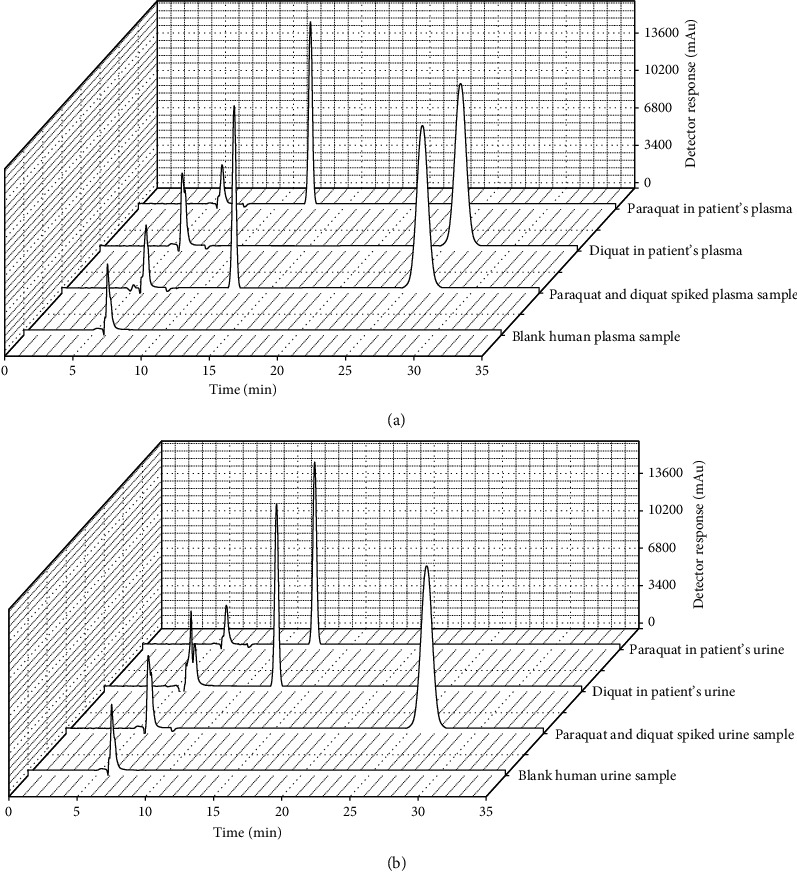
(a) The HPLC chromatogram of PQ and DQ in patient's plasma; PQ and DQ spiked into human plasma samples; and blank human plasma sample. (b) The HPLC chromatogram of PQ and DQ in patient's urine; PQ and DQ spiked into human urine samples; and blank human urine sample.

The method was applied to determine the concentrations of paraquat and diquat in patient plasma and urine (*n* = 6). The recovery was evaluated by the response signals of analytes in patient's plasma and urine with the response signals of spiked analytes in human plasma and urine samples. The matrix effect was evaluated by comparing the response signals of analytes in plasma and urine samples with those in neat solutions. As shown in Table 3, the recoveries of paraquat and diquat ranged from 87.5% to 98.7% with RSD less than 4.7%. The matrix effect of nine analytes ranged from 88.7% to 103.8% with RSD less than 4.5%, which is acceptable according to the FDA guidelines [35]. The HPLC chromatogram of PQ and DQ in patient's plasma and urine, the HPLC chromatogram of PQ and DQ spiked into human plasma samples, and the HPLC chromatogram of blank human plasma and urine are shown in Figure 15. The results showed that the developed method could effectively eliminate interference in plasma and urine samples.

## 4. Conclusion

In this work, a high selective and sensitive method for separation and determination of paraquat and diquat in human plasma and urine by magnetic dispersive solid phase extraction coupled with high-performance liquid chromatography was developed. The whole sample preparation time before determining by HPLC is dramatically decreased by using MDSPE without protein precipitation pretreatment. Therefore, the loss of trace analytes with the precipitation of matrix proteins, which results in a weak purification effect and low detection sensitivity and reliability, could be avoided. Desorption can also be completed in a few minutes by using NaOH solution and make the operation quick and easy. Meanwhile, the colorimetric detection method does not require high levels of financial investment or involve high instrument maintenance costs. This proposed that the naked-eye and quantitative detection methods have been successfully applied to detect PQ and DQ in real plasma and urine samples, demonstrating high sensitivity that allows for fast assessment of patient outcome based on routine clinical parameters following the history of PQ or DQ ingestion and thus facilitates the decision on treatment strategies.

## Figures and Tables

**Table 1:** Comparison of the developed method with other methods for determination of paraquat.

Sample	Method	Sample preparation	LOD (*μ*g/mL)	Linear range (*μ*g/mL)	RSD (%)	Recovery (%)	Ref.
Plasma and urine	GC-MS	RP-SPE^a^	0.05	0.1–5.0	—	98	[22]
Whole blood	HPLC/UV	RP-SPE	0.03	0.3–30.0	3.3	88–107	[32]
Plasma and urine	GC-MS	SPME^b^	0.01	0.1–5.0	5.1	94–100	[33]
Plasma	HPLC/UV	RP-SPE	0.01	0.02–1.0	3.6	98–107	[34]
Water, soil, and vegetables	HPLC/UV	RP-DLL ME^c^	0.02	—	<5.5	88–96	[14]
Plasma and urine	HPLC/UV	MSPE	0.004	0.028–0.57	2.3	90–108	This work

**Table 2:** The intraday and interday accuracy and precision of paraquat and diquat in human plasma and urine.

Sample	Analyte	Concentration (ng/mL)	Intraday			Interday		
			Mean (ng/mL)	Accuracy (%)	RSD (%)	Mean (ng/mL)	Accuracy (%)	RSD (%)
Plasma	Paraquat	100.0	90.6	90.6	5.7	84.9	84.9	4.7
		200.0	184.2	92.1	5.2	177.4	88.7	2.9
		500.0	482.0	96.4	3.4	463.5	92.7	2.6
	Diquat	100.0	88.9	88.9	5.4	84.5	84.5	4.6
		200.0	184.8	92.4	3.5	173.8	86.9	6.7
		500.0	485.5	96.7	3.8	465.5	92.7	5.2
Urine	Paraquat	100.0	89.5	89.5	4.7	85.4	85.4	6.3
		200.0	177.4	88.7	5.4	180.8	90.4	5.5
		500.0	467.2	93.4	2.7	450.5	90.1	3.9
	Diquat	100.0	88.6	88.6	5.8	84.6	84.6	5.1
		200.0	184.8	92.4	4.3	174.6	87.3	4.7
		500.0	477.5	95.5	4.6	456.5	91.2	3.8

**Table 3:** The recoveries and matrix effect of paraquat and diquat.

Sample	Analytes	Concentration (ng/mL)	Recoveries ± RSD (%)	Matrix effect ± RSD (%)
Patient's plasma	Paraquat	152.5	92.3 ± 2.6	88.7 ± 4.5
	Diquat	255.8	98.7 ± 3.2	95.7 ± 3.7
Patient's urine	Paraquat	210.6	87.5 ± 4.7	91.7 ± 2.5
	Diquat	356.8	93.5 ± 3.8	103.8 ± 4.3

## Data Availability

The data used to support the findings of this study are included within the article and are also available from the first author upon request.
